# Comprehensive Evaluation of Cultivated Land Quality at County Scale: A Case Study of Shengzhou, Zhejiang Province, China

**DOI:** 10.3390/ijerph17041169

**Published:** 2020-02-12

**Authors:** Yongzhong Tan, Hang Chen, Kuan Lian, Zhenning Yu

**Affiliations:** 1 Department of Land Management, School of Public Administration, Zhejiang University, Hangzhou 310058, China; tanyongzhong@zju.edu.cn (Y.T.); c.hang@zju.edu.cn (H.C.); kuanlizju@163.com (K.L.); 2 Land Academy for National Development, Zhejiang University, Hangzhou 310058, China; 3 School of Social and Public Administration, East China University of Science and Technology, Shanghai 200237, China

**Keywords:** land use, cultivated land quality, comprehensive evaluation, county scale

## Abstract

The existing evaluation system of cultivated land quality mainly considers the natural quality and utilisation conditions, but without sufficient emphasis on ecological environment, which can’t meet the requirements of the trinity pattern protection policy. This study, using GIS spatial analysis and multifactor comprehensive evaluation method, constructed a comprehensive evaluation index system, and applied it in Shengzhou. The results show that: (1) under the comprehensive evaluation system, the quality of cultivated land was classified into five levels and revealed normal distribution with the third level cultivated land area as the peak, successive reduction to the two poles, and the overall quality was good relatively; (2) A close relationship was observed between the quality grade of cultivated land and the landform, the valley plain with highest cultivated land quality was the main grain-producing areas. However, the cultivated land in mountainous areas was poor relatively, and vulnerable to geological disasters; (3) The quality grade of cultivated land was related to economical activities directly. The high-quality cultivated land resources made these towns the main grain-producing areas with many agricultural industries distribution. This study provided a new assessment approach that can support cultivated land grading, quality improvement, and sustainable usage, as well as providing a reference for related research and application.

## 1. Introduction

Cultivated land is the material basis for people to carry out agricultural production and plays an extremely important role in ensuring food security and stabilizing social order [1,2,3]. However, one-third of all agricultural land is considered either highly or moderately degraded now. Especially in China, since the Reform and Opening Up in 1978, the rapid development of urbanisation and industrialisation has transformed a large number of high-quality cultivated land into built-up land [4]. In order to protect the red line of 120 million hectares of cultivated land, the Chinese government has to develop low-quality land. The overall area has increased, but the quality and suitability declined [5,6,7]. Furthermore, environmental pollution and cultivated land reclamation have led to the degradation of the ecological environment. In addition, there is synergy between land degradation and two other major components of global environmental change (biodiversity and climate change). It is critical that land degradation is effectively addressed [8]. Therefore, a comprehensive understanding of cultivated land quality will have co-benefits for climate change mitigation and adaptation, and biodiversity conservation, in addition to enhancing food security and sustainable livelihoods [9].

The quality of cultivated land, that is, the land status and conditions [10]. Scholars used to consider soil fertility, climate and environment as the main indicators to evaluate the quality of cultivated land [11]. With the development of social economy, people’s perception about the concept and connotation has changed, not only the soil fertility, but the land suitability, potential productivity and ecological environment security [10]. The improvement of mechanisation has also brought attention to geographical location and infrastructure conditions.

The quality of cultivated land is the collection of multiple qualities, which are influenced by the composition and its combined characteristics [12]. Correspondingly, the cultivated land quality should be evaluated from the multi-level and multi-dimension, including soil conditions, site conditions, farming management, etc [9]. Meanwhile, research perspectives determine factors selection. When evaluating the potential for sustainable land use, the indicators are mainly focused on soil quality to indicate the basic soil fertility, soil environment and soil health [13]. When considering land suitability, besides natural indicators such as soil fertility that have a direct impact on cultivated land productivity, climate, and environmental factors will be used to select suitable species [14]. When farmers or managers evaluate the overall economics of the land, land tenure, utilisation conditions and landscape functions will be the core elements [15,16,17].

According to the current research, the understanding, theory and methods about cultivated land quality evaluation are deepening, reflecting the development of the era and the needs of the public continuously. However, the existing research on the evaluation of cultivated land quality mainly considers the natural quality and utilisation conditions, and the studies on the quality of ecological environment are insufficient, which can’t meet the requirements of the trinity pattern protection policy for the quantity, quality, and ecology of cultivated land. Although the consensus at present is that ecological quality should be considered in the evaluation index system, the specific index system on the ecological quality of cultivated land is still being explored continuously.

This paper, combining the cultivated land quality evaluation with the needs of the nation and the public, synthesized the research of the predecessors and national policy and selected the county administrative district of Shengzhou, Zhejiang Province as the research area, constructed a comprehensive evaluation index system from the three dimensions of natural quality, utilisation conditions and ecological security. The research evaluated the comprehensive quality of cultivated land under the needs of production efficiency, ecological security and sustainable use, and analysed the spatial distribution characteristics, influencing factors of cultivated land quality from the perspective of patches and towns. The evaluation system is constructed based on the principle of the systematics, representativeness, comprehensiveness and advisability, which reflects the public’s demand for diversification and the function versatility of the cultivated land comprehensively, and provides a theoretical basis for nation to implement the farmland protection policy. What’s more, it also provides reference for construction of the comprehensive evaluation index system of cultivated land quality and applicability in other analogous countries and regions.

## 2. Materials and Methods 

### 2.1. Study Area

**Figure 1:**
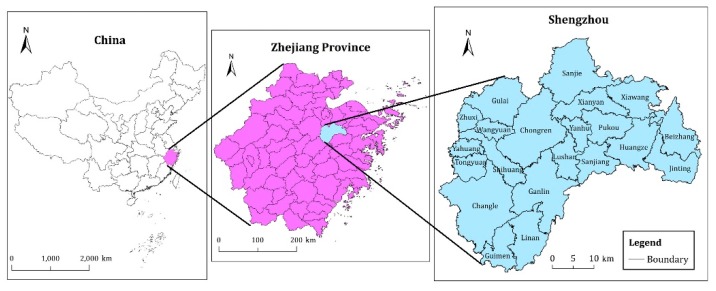
Location of the study area.

Shengzhou is a county-level city located in the eastern part of Zhejiang Province (Figure 1) and the upstream of Cao’e River, between 120°27′E to 121°06′E and 29°19′N to 29°49′N. This county belongs to the hilly mountainous area of eastern Zhejiang, with surrounding mountains, basins and plains in the middle. The landscape pattern is ‘seven tenths of mountains, one-tenth of water and two tenths of farmland’, which is similar to the characteristics of the whole Zhejiang Province. Shengzhou is a subtropical monsoon climate zone that is close to the southeast coast and affected by the alternating cold and warm air. The four seasons are distinct, the winter and summer are long and the spring and autumn are short. The climate is always humid and rainy and has the evident features of continental climate and basin microclimate. In 2015, the county’s cultivated land area was 45,127.49 ha and accounted for 25.22% of the total land area. The total grain output was 163,100 tons. Shengzhou is superior in ecological and environmental conditions and has a strong capacity in grain-production. However, in recent years, the improvement in urbanisation level, the adjustment of agricultural structure and the expansion of construction land have constantly changed the quality and pattern of cultivated land resources. Therefore, the quality of cultivated land resources should be assessed rationally to formulate and implement protection management programs scientifically for coordinating the contradiction of economic development and cultivated land protection. In the meantime, the social and economic development stage and the topography of Shengzhou are representative. Thus, this area was selected as the research site of this study.

### 2.2. Data Collection and Processing

The data in this work included (1) agricultural land classification database of Shengzhou in 2015 for generating index attributes, such as altitude, surface soil texture, soil organic matter, irrigation guarantee rate, slope, soil pH and tillage layer thickness; (2) the land use map of Shengzhou in 2015 (1:10,000) for obtaining current data, such as highways and administrative centres; and (3) Geospatial data cloud (http://www.gscloud.cn) for generating NDVI data of Shengzhou in 2015.

On the basis of the land use map of Shengzhou in 2015 (1:10,000), this study used cultivated land map patches of 45,655 (excluding sporadic cultivated land) in the 2015 Shengzhou Land Change Survey as the evaluation unit. At the same time, we utilised ArcGIS (developed by ESRI in Redlands, CA, US) to realise the projection transformation and vectorisation of each factor. On this basis, the factor method was used to calculate the quality score of each evaluation unit.

### 2.3. Methods

#### 2.3.1. Comprehensive Evaluation Index System of Cultivated Land Quality and Index Weight

“The opinions on strengthening farmland protection and improving the balance between occupation and compensation of farmland” by the Central Committee of the Communist Party of China and the State Council suggest that we must vigorously strengthen the protection of quantity, quality and ecology of cultivated land and build a trinity food security pattern. However, the “Guidelines for the Grading of Cultivated Land Quality and Agricultural Land Evaluation in Zhejiang Province in 2016” [18], which assessed the cultivated land quality based on the fertility and environmental quality, selected the landform, slope, soil texture, tillage layer thickness, PH, soil organic matter, irrigation guarantee rate and the degree of soil pollution as indicators to construct the evaluation system. Besides that, the “Regulations for classification on agriculture land” [19] selected the impact degree of centre town, road accessibility, regularity of plots and so on to build the evaluation system from three dimensions of natural factors, socio-economic factors and location factors. The natural quality and utilisation conditions are the basis of high yield and efficiency, and also the main aspects of the evaluation of cultivated land quality [20]. However, the comprehensive evaluation of cultivated land quality must consider the ecological factors [21,22], which can characterise the sustainability of cultivated land use in some ways. Based on the research of relevant scholars, in addition to considering slope and NDVI of cultivated land, which have potential impacts on soil erosion [23], this system should also include farmland connectivity that threatens species diversity [24,25,26]. Above all, this paper, on the basis of national standards and related research, used the factor method to construct the comprehensive evaluation index system of cultivated land quality from three aspects, namely, natural quality, utilisation conditions and ecological security. 

Index grading and assignment rules were based on the “Guidelines for the Grading of Cultivated Land Quality and Agricultural Land Evaluation in Zhejiang Province in 2016”, “Regulations for classification on agriculture land” and “Technical specifications for assessment and rating criteria of cultivated land quality” [27]. In view of this background, we firstly used the analytic hierarchy process to determine the weight of each index. Then, the expert scoring method was utilised to re-determine the weight of each index (Table 1). Nine experts in relevant field from the Zhejiang Academy of Agricultural Sciences, Provincial Land Consolidation Centre and Zhejiang University and Zhejiang University of Finance and Economics scored the indicator weight table, and they are all well-known in the field and have rich experience. Lastly, the weight of each evaluation index was determined.

As can be seen from Table 1, the natural quality, utilisation conditions, and ecological security weights of the evaluation system are 0.45, 0.32, and 0.23, which are in line with the actual situation of cultivated land use. The main use of cultivated land is to produce food, and it must be ensured that the cultivated land has a certain degree of productivity, which reflects the superiority of its natural quality. On this basis, it is wise that optimizing the utilisation conditions of cultivated land, by increasing investment, to achieve high efficiency and yield. Finally, in order to achieve sustainable use and health of cultivated land, its ecological security is gradually achieving emphasis, and it should occupy a certain weight in the system. Because the cultivated land is not polluted here, and the existing forms in mountainous areas are mainly terrace. There are no fatal indicators that affect the quality of cultivated land. Therefore, the weight of each indicator is between 0.07 and 0.1, and the difference is not distinct.

#### 2.3.2. Standardisation of Comprehensive Evaluation Indicators for Cultivated Land Quality

In view of the differences in these evaluation indicators, the closed interval of [0,100] was adopted to realise the conversion from the influencing degree of each index to the comprehensive evaluation score of quality, which indicates how much an index works on the evaluation result. The standardisation of each indicator was based on the “Guidelines for the Grading of Cultivated Land Quality and Agricultural Land Evaluation in Zhejiang Province in 2016”, “Regulations for classification on agriculture land” and “Technical specifications for assessment and rating criteria of cultivated land quality”.

(1) Assessment of the natural quality of cultivated land

The natural quality of cultivated land is an important condition to evaluate land productivity and provides the basis for determining the production capacity. As the cropping system and climatic conditions are the same in the county area, the main factors affecting the quality of cultivated land are soil fertiliser and soil texture. In addition, a big difference was observed with respect to the altitude of cultivated land. Therefore, the altitude was included as a natural quality indicator in the evaluation system. In accordance with the idea of assessing the natural quality of cultivated land in agricultural land classification and the real situation of Shengzhou, five indicators, namely, tillage layer thickness, altitude, soil texture, soil organic matter, and soil pH, were selected to characterise the natural quality of cultivated land. The quantitative values of the above-mentioned indicators were all derived from the agricultural land classification database of Shengzhou in 2015.

(2) Evaluation of cultivated land utilisation conditions

Utilisation conditions were used to characterise the availability and convenience of the cultivated land resources in the region. The farming environment and location will influence the use of land to a certain degree. Therefore, the irrigation guarantee rate, road accessibility and regularity of plots were selected to reflect the advantages and disadvantages of the farming environment. The impact degree of centre town was selected to quantitatively analyse the location of cultivated land.

1) Irrigation guarantee rate. The irrigation guarantee rate is an important indicator to measure the quality of cultivated land. In addition, the irrigation guarantee rate indicates the possibility that the expected amount of water can be fully satisfied in the continuous irrigation and is closely related to the distribution of irrigation facilities. The quantified value of its qualitative index was derived from the agricultural land classification database of Shengzhou in 2015.

2) Road accessibility. Road accessibility indicates the convenience of transportation of agricultural equipment to the project area, and belongs to the linear indicator. A road that is near and wide is favourable for the introduction of agricultural machinery. The current road was assigned by the linear attenuation method in the “Regulations for classification on agriculture land”. The formula used for road accessibility is the following (1):

(1) $$f_{i}{=M}_{i\;}\left(1-r\right),$$

In the formula, *f_i_* is the effect value of each road, *M_i_* is the functional score of a certain road and *r* is the relative distances.

3) The impact degree of centre town. The index indicates the indirect influence of the central town on the land use efficiency and spatial layout of each unit, and belongs to the point indicator. This indicator was assigned by the exponential attenuation method in the “Regulations for classification on agriculture land”. The formula used for the index is the following (2):

(2) $$f_{i}{=M}_{i}\;(1-R),$$

In the formula, *f_i_* is the effect value of each level, *M_i_* is the functional score of a certain level and *R* is the relative distance.

4) Regularity of plots. The rule of the plot shape greatly influences agricultural mechanisation and cultivated land management. The index for measuring the regularity of plots can be expressed by the fractal dimension (FRAC) in landscape ecology. The theoretical value ranges between 1.0 and 2.0, and the larger the index is, the more irregular the shape is. The formula used for the index is the following (3):

(3) $$FRAC=\frac{2\log(p/4)}{\log(\alpha )},$$

In the formula, *FRAC* is the regularity of the field; *p* is the plot perimeter of the plot(m); and *α* is the plot area(m^2^).

(3) Evaluation of cultivated land ecological quality

Cultivated land ecological quality is the indicator of ecological tolerance in cultivated land use, that is, the ability to prevent soil erosion and exert ecological value under different external environments [28]. At the same time, the concentration and contiguity of cultivated land are important for improving the production capacity and increasing the value of agricultural land and can also avoid the loss of biodiversity and the degradation of ecosystem caused by fragmentation of cultivated land landscape. In this study, the terrain slope, NDVI value, and farmland connectivity were used to reflect the ability of soil and water conservation, water conservation and ecological services. At the same time, the farmland connectivity was utilised to analyse the fragmentation degree of cultivated land.

1) Slope. The slope is closely related to soil fertility, soil and water conservation and mechanisation level. It is the main factor reflecting the soil and water conservation capacity of cultivated land and promoting the sustainable and stable ecological quality of cultivated land [29]. The quantified value of its qualitative index was derived from the agricultural land classification database of Shengzhou in 2015.

2) NDVI. NDVI value indicates the normalised vegetation index, which can reflect the ecological indicators, such as vegetation coverage, and is an important indicator for measuring the cultivated land ecosystem services. The NDVI data source of this study adopted remote sensing image of Landsat-8 30 m resolution, selected the image data in August 2015 and during the lush period of rice growth and calculated the maximum of the vegetation NDVI in August of Shengzhou. ArcGIS was also used to obtain the NDVI value of each unit and the maximum value. On this basis, all the NDVI values of the evaluation unit were divided into five intervals by the Natural Breakpoint Method (NBM). A large NDVI value indicates good vegetation coverage and high ecological quality.

3) Farmland connectivity. In landscape ecology, farmland connectivity is important for ecological species protection and habitat selection. To date, many studies have discussed the issue of farmland connectivity. This indicator is expressed by the farmland connectivity *T*. It is a quantification of the plot size. After the statistics of the total cultivated land area in the study area, the threshold s of the plot area in the formula was determined by the NBM. A large *T* value indicates high connectivity degree. The formulas used for the index is the following (4):

(4) $$T=\{\begin{array}{cc} 40 & s\leq 0.3\mathrm{ha}\\ 40+60\frac{s-0.3}{4.0-0.3} & 0.3\mathrm{ha}<\;s\;<4.0\;\mathrm{ha},\\ 100 & s\geq 4.0\mathrm{ha} \end{array}$$

In the formula, *T* is the connectivity of the cultivated land and *s* is the area of the plot.

#### 2.3.3. Comprehensive Evaluation Model of Cultivated Land Quality

After the standardisation of each index, Weighted Sum Method (WSM) was used according to the weight of each index’s impact degree on the quality of cultivated land. The comprehensive evaluation score of the evaluation unit was also calculated by ArcGIS as the following (5):

(5) $$H_{i}=\sum_{i=1}^{n}w_{j}f_{\mathrm{ij}},$$

where *H_i_* is the comprehensive evaluation score of the *i_th_* evaluation unit; *w_j_* is the weight of the *j_th_* evaluation index; and *f_ij_* is the standardised value of the *j_th_* evaluation index of the *i_th_* evaluation unit.

After comprehensive evaluation of cultivated land quality, we applied the area weighting factor method and the total fractional frequency curve method based on patch scores to get the index value of each town and classify the cultivated land quality. Those methods are simple and have a wide range of applications, and not specific for this research, so they are not described in detail in this section.

## 3. Results

### 3.1. Comprehensive Evaluation Results of Cultivated Land Quality

**Figure 2:**
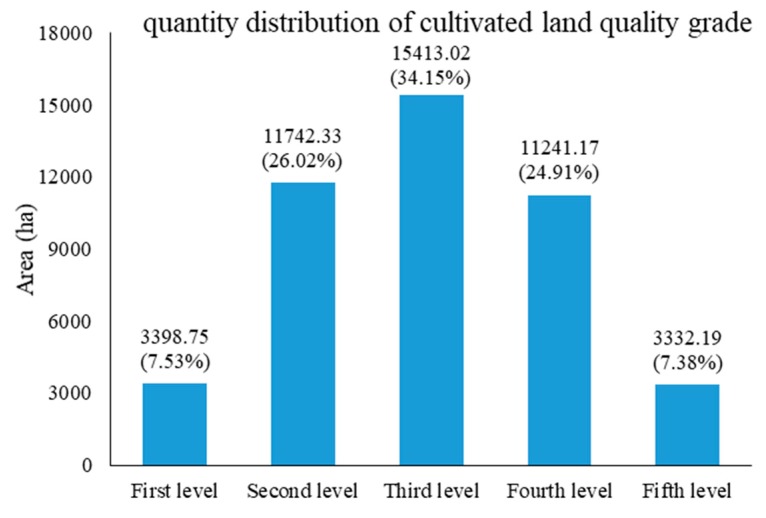
Quantity distribution of cultivated land quality grade in the study area.

The comprehensive score of cultivated land quality in Shengzhou was between 48.20 and 97.40 by area weighting factor method, and the average comprehensive score of the whole county was 78.08, which means that the overall quality of cultivated land was good. The total fractional frequency curve method was used to divide the cultivated land into five levels. Amongst them, those less than 66.50 were the fifth level, between 66.50 and 76.00 were the fourth, between 76.00 and 85.00 were the third, between 85.00 and 90.00 were the second and those greater than 90.00 or equal to 90.00 were the first level. The results of the quantity distribution showed that the quality of cultivated land in the study area revealed normal distribution, of which the third level land area was the largest, followed by the second and fourth levels. The first and fifth levels were the least (Figure 2).

According to the results, the area of the third level land was the largest and accounted for 34.15%. The following were the second level and fourth level land and accounted for 26.02% and 24.91% of the total cultivated land area, respectively. The area of the fifth level land was less and accounted for 7.38% of the total area, followed by the first level land that accounted for 7.53%. Overall, the large proportion of the cultivated land in the study area was at the upper-middle level, with great productive potential. This result generally indicated that the quality of cultivated land in Shengzhou was at a relatively high level.

### 3.2. Regional Distribution of Cultivated Land Quality

**Figure 3:**
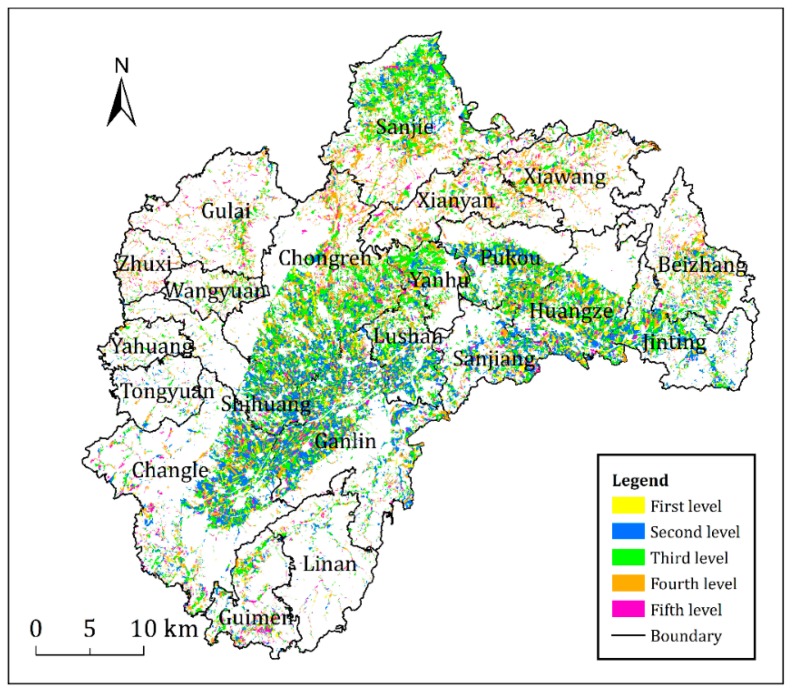
Distribution of the quality of cultivated land in the study area.

From the distribution of cultivated land at all levels (Figure 3), the first and second level land areas were only distributed in the valley plain cultivated area in the middle and the plain cultivated areas in the north. In addition, the main landform was the valley plain. The first and second level land areas, which were cultivated land with the highest comprehensive productive potential in Shengzhou, had high evaluation index values, such as flat ground, good production performance and strong ability of drainage or drought resistance. The cultivated layer was relatively thick, and soil texture of the tilth soil was mainly loam, with a few of silt loam and clay loam. The soil had high nutrient content, good water retention and fertiliser retention performance. No limiting factors were observed in cultivation. This condition ensured high-yield and stable farmland whether in drought or flood.

The third level land area was mainly distributed in low-slope hilly land, the edge of the valley basin in the middle and lower hills and ridges in the north, which was the most common level in Shengzhou. The third level land area was a type of transition from plain to mountain, wherein the cursor attribute was between the two. The soil layer was relatively deep, and soil texture was mainly silt loam and clay loam, wherein the organic matter content was generally high, and irrigation and drainage system were relatively perfect. In addition, the third level cultivated land had strong ability of drought resistance and drainage, good planting suitability and ability of harvesting whether in drought or flood. This level of cultivated land was suitable for crop growth, which was the largest and good-quality grain production area in Shengzhou. The quality of cultivated land was mainly affected by the soil nutrient content.

The fourth level land area also occupied a relatively high proportion in the cultivated land area of Shengzhou and was mainly distributed in the northern low-hill gentle slopes, the eastern ridges, terraces and intersected with the fifth level land area. Most of the landform types were low-hill platforms and low-altitude hillsides. The slope of the land was slightly large, and the ability of drought resistance and drainage was general. The soil texture of the cultivated layer was mainly clay, and the soil was generally acidified. The soil lacked nutrient, and the crop yield level was stable but insufficiently high due to the relatively thin cultivated layer and poor fertiliser-preserving ability. The limiting factors were mainly the landform type, soil pH, irrigation and drainage conditions and soil nutrient. For this type of cultivated land area, strengthening the soil management, applying alkaline fertiliser and preventing soil erosion were necessary.

The fifth level land area was least, mainly concentrated in the northwest low-hill rock platforms and intersected with the fourth level land area. The fifth level land area belonged to the mountain level type. The altitude of the cultivated land was approximately 500–800 m, where its sunshine was less than that in the plain, and production capacity was relatively poor. The slope of the ground was relatively large, and the cultivated layer was relatively thin. The soil texture was mainly clay except partial sand, and the soil acidification was relatively serious. The soil nutrient status was generally at the middle-lower level, and the difference was distinct. What’s more, no irrigation conditions were observed practically, and most of them were fields on hilltops that depend on rains for water. The quality of cultivated land at this level was mainly affected by the landform type, soil pH, slope, road accessibility, soil nutrient, and irrigation guarantee rate. Therefore, the land was difficult to be improved and utilized relatively.

In general, the cultivated land quality of the land area in Shengzhou’s valley plain was the highest, and its production capacity was the largest, followed by the low-hill land area and the mountainous area. Plains, hills and mountainous areas nearly distribute crosswise. The topography and landform generally determined the features of the cultivated land quality. A close relationship was observed between the cultivated land quality level and the landform. At the same time, the natural quality, such as soil texture, soil organic matter and soil PH, had a great impact on grain-producing.

### 3.3. Administrative Distribution Law of Cultivated Land Quality

From the administrative division of towns, great differences were observed in the cultivated land quality level (Table 2). The first level land area was mainly concentrated in Chongren, Ganlin, Changle, and Huangze and accounted for 57.09%. The second level land area was mainly distributed in Chongren, Ganlin, and Changle and accounted for 12.05%, 19.07%, and 14.10%, respectively. The third level land area was mainly distributed in Chongren, Ganlin, and Sanjie and accounted for 15.78%, 10.75%, and 14.72%, respectively. The fourth level land area was distributed in all towns and was relatively scattered. Amongst which, Chongren and Sanjie had the highest proportions and accounted for 15.99% and 12.91%, respectively, whereas the other towns all accounted for less than 10%. The fifth level land area was mainly concentrated in Chongren, Ganlin, and Changle and accounted for 12.05%, 12.81%, and 13.85%, respectively. Notably, the cultivated land quality was also quite different in each town. However, the cultivated land quality of Ganlin, Chongren, Changle and Huangze in the valley plain area was generally good. Most of the cultivated land was river valley plain or basin. The cultivated land quality of Zhuxi, Beizhang, Xiawang, and Xianyan was generally poor, and the landform types were all hilly hillsides or low-hill terraces, which were unsuitable for developing crops. 

The results combined with the regional economic development status of the study area indicate that the economic industries in the regions with preferential cultivated land quality are mainly agricultural production. They are generally the main grain-producing areas in the regions and have good natural conditions and relatively perfect infrastructure for serving agricultural production. The towns with poor cultivated land quality are mainly distributed in mountainous areas or semi-mountainous areas. Due to the poor soil conservation conditions, soil erosion is prone to occur, resulting in a decline in soil quality. From the perspective of regional distribution of cultivated land quality and the economic development level of the towns, the cultivated land quality directly impacts the local economic development level and the features of agricultural activities. Therefore, the regional differences in the cultivated land quality have also caused the imbalance of economic development in Shengzhou.

## 4. Discussion

### 4.1. Construction of Cultivated Land Evaluation System

The quality of cultivated land includes soil quality, environmental quality, and management quality [20]. “The opinions on strengthening farmland protection and improving the balance between occupation and compensation of farmland” by the Central Committee of the Communist Party of China and the State Council suggest that we must vigorously strengthen the protection of quantity, quality and ecology of cultivated land and build a trinity food security pattern. However, there is no consistent evaluation system at the current research. This paper constructed a comprehensive evaluation system from the natural quality, utilisation conditions and ecological security of cultivated land, to characterise the versatility in the ecosystem.

According to the land natural attribute, this paper selected the tillage layer thickness, altitude, soil texture, soil organic matter and soil pH to indicate the natural conditions. The tillage layer thickness is often used as an important indicator to judge land degradation for farmers. The soil is a non-renewable resource with ecological functions to maintain and improve soil and water conservation [30,31]. The core of cultivated land quality is soil quality. Soil organic matter and pH have been used for soil quality evaluation [32,33,34,35]. Soil organic matter is the basis of land fertility [36,37]; pH is one of the most important parameters, and most crops develop best with a pH of 5.5 to 6.5 [38]; altitude and soil texture are selected for agricultural suitability evaluation.

The Land Evaluation and Site Analysis System (LESA), developed by the United States Department of Agriculture in 1980, emphasizes that cultivated land conditions play an important role in agricultural land quality evaluation [39]. Farmers agree that stable utilisation conditions are one of the representative characteristics of high-quality cultivated land [40]. In this paper, the irrigation guarantee rate, road accessibility, the impact degree of centre town and regularity were selected as indicators of comprehensive utilisation conditions. Water is the most critical factor affecting food production except soil [41,42]. A reasonable irrigation model not only improves irrigation efficiency, but also maintains water and soil balance in farmland [43]. Accessibility is considered to be one of the most important factors affecting land use [44]. People combine land accessibility with land use spatial patterns as an important basis for distinguishing farming practices [45]. It is usually calculated through topographic maps or represented by the distance from land to road or market [46,47], and the impact of accessibility on land use is expressed by road accessibility and the impact degree of centre town. Human activities shape the natural landscape into a regular rectangular with distinct edges and corners, especially the agricultural land, and different sizes of plots form a checkerboard shape [27,48,49], a large number of studies have shown that agricultural land regulation can help improve land use efficiency [50,51,52].

In the past ten years, China′s agriculture development was in the transition period from the productivism to the post-productivism. From pursuing production increase to paying more attention to land quality, safety, efficiency and ecology [53]. In order to reflect the ecological quality, ecological factors must be adopted as an important indicator in evaluation [20,21]. The ecological quality of cultivated land, acting as an ecological subsystem, mainly refers to the status of ecological functions, such as soil and water conservation, water conservation, air purification, climate regulation and biodiversity conservation. Based on the previous research, this paper selected indicators such as slope, NDVI and farmland connectivity to measure the ecological quality. The topographic slope is the main factor reflecting the soil and water conservation capacity of cultivated land and promoting the sustainable and stable ecological quality of cultivated land [22]. NDVI can reflect the ecological indicators and has been used as an important indicator for estimating crop yields, monitoring land degradation [23]. Land fragmentation not only hinders agricultural modernisation and mechanisation, but is also considered to be one of the important factors threatening species diversity and causing land degradation, so the farmland connectivity is important for the protection of ecological species and the selection of habitat [24,25].

### 4.2. The Impact of Research Scale on Selection of Indicators

The indicators of cultivated land quality evaluation system should characterise the versatility of cultivated land, not only reflect the comprehensive effects of biological, physical and chemical properties of the soil, but also consider the ecosystem outside. However, the traditional evaluation of cultivated land quality only selects the physical and chemical characteristics of soil as indicators. The ecological status of cultivated land is considered insufficiently [54]. Except that, there are also differences in different research scales. 

At a national or provincial scale, the cultivated land quality evaluation system is usually constructed based on natural conditions, land use and input-output levels. This type of data is basically based on prefecture-level cities and extracted by statistical data or remote sensing. That is easy to get. When evaluating the quality of cultivated land in grain bases or prefecture-level administrative area, the physical and chemical properties of soil, farming conditions, health status and biological characteristics are the main criteria. Soil indicators are mostly based on on-site surveys. Land conditions were obtained by remote sensing. When cultivated land quality changes caused by land consolidation projects, the evaluation system consists of indicators such as soil quality, field size, and flatness. It needs high-density sampling data to ensure data integrity and credibility. The spatial land quality obtained through geo-statistics has certain reference, but the rapid development of remote sensing provides potential for evaluating. It’s an important way to evaluate land quality by combing experiment with field survey and remote sensing [36].

The county administrative region is the most important unit in China’s land administration and land use, and can control land use based on the evaluation results directly. This paper, taking Shengzhou as research area, constructed the cultivated land quality evaluation system from the three dimensions of natural quality, utilisation conditions and ecological security, and selected the core indicators in social production and landscape ecology from macroscopic and microscopic. Reflecting the public′s demand diversification and the function versatility for the cultivated land comprehensively, it can provide a theoretical basis for the nation to implement the farmland protection policy, and reference for comprehensive management of cultivated land in Shengzhou. More importantly, the system has certain applicability in the evaluation of cultivated land quality in other analogous countries and regions.

### 4.3. Research Prospect

During actual production, the selected participation factors, evaluation criteria and weight determination will directly impact the comprehensive evaluation results of cultivated land quality due to the complex factors affecting the cultivated land quality. With the advancement of technology, the advancement of technology, it is necessary to construct a scientific comprehensive evaluation index system for cultivated land quality. In addition, the evaluation carried out in this study is the quality of cultivated land at one point in time. The utilisation conditions and ecological security will change with the development of economy and society. Therefore, the evaluation and analysis of the temporal and spatial dynamics of cultivated land quality will be the focus of future research by RS and GIS.

## 5. Conclusions

The evaluation of cultivated land quality is the basis of relevant studies, such as the delineation of permanent basic farmland, and also has important significance for guiding the practices, including assessment of cultivated land occupation and supplementary balance and land reclamation. Based on RS, GIS, and social statistics, this paper constructed a comprehensive quality evaluation system, and evaluated the cultivated land quality in Shengzhou.

The evaluation results reveal that the average comprehensive score of cultivated land quality in Shengzhou was 78.08, and the overall quality was good. The distribution of each level showed a normal distribution with the third level cultivated land area as the peak and successive reduction to the two poles. The proportion of cultivated land quality above the third level was 67.71%. This result indicated that the quality of cultivated land in Shengzhou was at a high level relatively. A close relationship was observed between the quality grade of the cultivated land and the landform type, soil pH, irrigation and drainage conditions and soil nutrient. The geographical distribution of the cultivated land quality was quite evident. The third level cultivated land area or above was mainly distributed in the middle low hill, valley plain and northern plain area. The soil type was mainly loam, and the cultivation was unrestricted. The land in high altitude mountainous areas had relatively thin cultivated land layer. The terrain changed greatly and was vulnerable to geological disasters. Thus, the development potential was low. The third level cultivated land area or above was mainly distributed in the valley plains, such as Chongren, Ganlin, Changle, and Huangze. The high-quality cultivated land resources made these towns the main grain-producing areas of Shengzhou. This situation had an important influence on the features of regional agricultural activities and the distribution of agricultural industry. 

To a certain degree, the evaluation index system constructed in this paper has systematicness and representativeness, which can reflect the public′s demand diversification and the function versatility for the cultivated land. This study provided a new assessment approach, which can support cultivated land grading, quality improvement, and sustainable usage, and other analogous countries and regions can take the evaluation system constructed in this paper as a reference.

## Figures and Tables

**Table 1:** Comprehensive evaluation indicator system and quantification standards of cultivated land quality.

Criteria	Index	Classification Standard								Index Weight
		100	90	80	70	60	50	40	30	
Natural quality	Tillage layer thickness/cm	≥100		80–100		60–80		30–60	<30	0.08
	Altitude/m	<50	50–150	150–300	300–500		500–800		≥800	0.09
	Soil texture	loam	silt loam /clay loam		loamy clay/sandy loam		clay		sand	0.10
	Soil organic matter/(g/Kg)	≥30	25–30	20–25	15–20	10–15		5–10	<5	0.08
	Soil pH	6.0–7.0	5.8–6.0 /7.0–7.2	5.5–5.8 /7.2–7.5		5–5.5 /7.5–8		<5/ >8		0.10
Utilisation conditions	Irrigation guarantee rate	≥70		50–70		30–50			<30	0.08
	Road accessibility	≥2.5		2.0–2.5		1.5–2.0			<1.5	0.09
	The impact degree of centre town	≥70.5	58.0–70.5			45.5–58.0			<45.5	0.08
	Regularity of plots	≤1.02		1.02–1.06		1.06–1.10		1.10–1.50	>1.50	0.07
Ecological security	Slope	0–2		2–6		6–15			>15	0.08
	NDVI	>0.45/ <0		0.35–0.45		0.25–0.35		0–0.25		0.08
	Farmland connectivity	>80		50–80		40–50		40		0.07

**Table 2:** Quality grade statistics of cultivated land in various towns in the study area.

Towns	First Level		Second Level		Third Level		Fourth Level		Fifth Level		Index Value
	Area/ha	PCT/%	Area/ha	PCT/%	Area/ha	PCT/%	Area/ha	PCT/%	Area/ha	PCT/%	
Beizhang	71.62	2.11	316.81	2.70	552.38	3.58	583.90	5.19	144.98	4.35	72.43
Chongren	442.23	13.01	1414.58	12.05	2432.36	15.78	1798.03	15.99	401.66	12.05	79.76
Ganlin	586.67	17.26	2239.12	19.07	1656.90	10.75	1054.52	9.38	426.80	12.81	80.41
Gulai	11.26	0.33	64.27	0.55	265.40	1.72	360.89	3.21	163.95	4.92	76.72
Guimen	59.01	1.74	178.20	1.52	306.81	1.99	250.34	2.23	175.40	5.26	75.73
Huangze	392.76	11.56	1034.47	8.81	1349.19	8.75	873.55	7.77	153.57	4.61	79.90
Jinting	199.92	5.88	639.02	5.44	640.86	4.16	271.53	2.42	84.50	2.54	78.04
Linan	15.38	0.45	96.12	0.82	131.54	0.85	104.36	0.93	55.46	1.66	76.35
Lushan	237.38	6.98	760.27	6.47	766.86	4.98	378.02	3.36	121.80	3.66	76.53
Pukou	212.22	6.24	677.29	5.77	724.65	4.70	444.02	3.95	66.89	2.01	77.43
Sanjiang	166.59	4.90	622.98	5.31	536.11	3.48	365.14	3.25	176.79	5.31	78.49
Sanjie	223.94	6.59	836.69	7.13	2268.24	14.72	1451.63	12.91	282.39	8.47	77.39
Shihuang	212.50	6.25	713.93	6.08	510.41	3.31	277.55	2.47	105.32	3.16	78.25
Tongyuan	2.90	0.09	69.86	0.59	68.46	0.44	64.81	0.58	20.81	0.62	77.41
Wangyuan	4.97	0.15	51.43	0.44	143.36	0.93	144.88	1.29	37.14	1.11	75.22
Xiawang	8.16	0.24	57.53	0.49	402.76	2.61	733.20	6.52	163.52	4.91	72.52
Xianyan	13.14	0.39	85.02	0.72	437.09	2.84	695.25	6.18	171.14	5.14	73.03
Yahuang	3.63	0.11	24.60	0.21	82.34	0.53	69.70	0.62	15.03	0.45	76.24
Yanhu	15.83	0.47	194.27	1.65	616.43	4.00	306.21	2.72	39.75	1.19	77.65
Changle	518.64	15.26	1655.22	14.10	1459.91	9.47	879.45	7.82	461.41	13.85	79.66
Zhuxi	—	0.00	10.65	0.09	60.96	0.40	134.22	1.19	63.88	1.92	70.83
Total	3398.75	100.00	11742.33	100.00	15413.02	100.00	11241.17	100.00	3332.19	100.00	78.08

## References

[1] Foley J.A., Ramankutty N., Brauman K.A., Cassidy E.S., Gerber J.S., Johnston M., Mueller N.D., O’Connell C., Ray D.K., West P.C. (2011). Solutions for a cultivated planet. Nature.

[2] Francis C.A., Hansen T.E., Fox A.A., Hesje P.J., Nelson H.E., Lawseth A.E., English A. (2012). Farmland conversion to non-agricultural uses in the US and Canada: Current impacts and concerns for the future. Int. J. Agric. Sustain..

[3] Chai J., Wang Z., Yang J., Zhang L. (2019). Analysis for spatial-temporal changes of grain production and farmland resource: Evidence from Hubei Province, central China. J. Clean. Prod..

[4] Kuang W., Liu J., Dong J., Chi W., Zhang C. (2016). The rapid and massive urban and industrial land expansions in China between 1990 and 2010: A CLUD-based analysis of their trajectories, patterns, and drivers. Landsc. Urban Plan..

[5] Xu X., Wang L., Cai H., Wang L., Liu L., Wang H. (2017). The influences of spatiotemporal change of cultivated land on food crop production potential in China. Food Secur..

[6] Song W., Pijanowski B.C. (2014). The effects of China’s cultivated land balance program on potential land productivity at a national scale. Appl. Geogr..

[7] Kong X. (2014). China must protect high-quality arable land. Nat. News.

[8] Xiao Q., Zong Y.T., Lu S.G. (2015). Assessment of heavy metal pollution and human health risk in urban soils of steel industrial city (Anshan), Liaoning, Northeast China. Ecotoxicol. Environ. Safe.

[9] Liu Y., Zhang Y., Guo L. (2010). Towards realistic assessment of cultivated land quality in an ecologically fragile environment: A satellite imagery-based approach. Appl. Geogr..

[10] Jiang G., Zhang R., Ma W., Zhou D., Wang X., He X. (2017). Cultivated land productivity potential improvement in land consolidation schemes in Shenyang, China: Assessment and policy implications. Land Use Policy.

[11] Song W., Wu K., Zhao H., Zhao R., Li T. (2019). Arrangement of high-standard basic farmland construction based on village-region cultivated land quality uniformity. Chin. Geogr. Sci..

[12] Dumanski J., Pieri C. (2000). Land quality indicators: Research plan. Agric. Ecosyst. Environ..

[13] Tesfahunegn G.B. (2016). Soil quality indicators response to land use and soil management systems in northern Ethiopia’s catchment. Land Degrad. Dev..

[14] Vasu D., Srivastava R., Patil N.G., Tiwary P., Chandran P., Singh S.K. (2018). A comparative assessment of land suitability evaluation methods for agricultural land use planning at village level. Land Use Policy.

[15] Ma L., Bo J., Li X., Fang F., Cheng W. (2019). Identifying key landscape pattern indices influencing the ecological security of inland river basin: The middle and lower reaches of Shule River Basin as an example. Sci. Total Environ..

[16] Teshome A., de Graaff J., Ritsema C., Kassie M. (2016). Farmers’ perceptions about the influence of land quality, land fragmentation and tenure systems on sustainable land management in the north western Ethiopian highlands. Land Degrad. Dev..

[17] Coyle C., Creamer R.E., Schulte R.P., O’Sullivan L., Jordan P. (2016). A functional land management conceptual framework under soil drainage and land use scenarios. Environ. Sci. Policy.

[18] Land Consolidation Centre of Zhejiang Province (2016). Guidelines for the Grading of Cultivated Land Quality and Agricultural Land Evaluation in Zhejiang Province in 2016.

[19] General Administration of Quality Supervision, Inspection and Quarantine of the People’s Republic of China, Standardisation Administration of the People’s Republic of China (2012). Regulations for Classification on Agriculture Land of the People’s Republic of China.

[20] De la Rosa D., Mayol F., Diaz-Pereira E., Fernandez M., de la Rosa D. (2004). A land evaluation decision support system (MicroLEIS DSS) for agricultural soil protection: With special reference to the Mediterranean region. Environ. Model. Softw..

[21] Feng Y., Yang Q., Tong X., Chen L. (2018). Evaluating land ecological security and examining its relationships with driving factors using GIS and generalized additive model. Sci. Total Environ..

[22] Wilson G.A. (2009). The spatiality of multifunctional agriculture: A human geography perspective. Geoforum.

[23] Nabahungu N.L., Visser S.M. (2013). Farmers’ knowledge and perception of agricultural wetland management in Rwanda. Land Degrad. Dev..

[24] Karnieli A., Qin Z., Wu B., Panov N., Yan F. (2014). Spatio-temporal dynamics of land-use and land-cover in the Mu Us sandy land, China, using the change vector analysis technique. Remote Sens..

[25] Sklenicka P. (2016). Classification of farmland ownership fragmentation as a cause of land degradation: A review on typology, consequences, and remedies. Land Use Policy.

[26] Pineda E., Halffter G. (2004). Species diversity and habitat fragmentation: Frogs in a tropical montane landscape in Mexico. Biol. Conserv..

[27] Quality and Technology Supervision of Zhejiang Province (2013). Technical Specifications for Assessment and Rating Criteria of Cultivated Land Quality.

[28] Bhardwaj A.K., Jasrotia P., Hamilton S.K., Robertson G.P. (2011). Ecological management of intensively cropped agro-ecosystems improves soil quality with sustained productivity. Agric. Ecosyst. Environ..

[29] Ren T., Wang J., Chen Q., Zhang F., Lu S. (2014). The effects of manure and nitrogen fertilizer applications on soil organic carbon and nitrogen in a high-input cropping system. PLoS ONE.

[30] Peerawat M., Blaud A., Trap J., Chevallier T., Alonso P., Gay F., Thaler P., Spor A., Sebag D., Choosai C. (2018). Rubber plantation ageing controls soil biodiversity after land conversion from cassava. Agric. Ecosyst. Environ..

[31] Nabiollahi K., Golmohamadi F., Taghizadeh-Mehrjardi R., Kerry R., Davari M. (2018). Assessing the effects of slope gradient and land use change on soil quality degradation through digital mapping of soil quality indices and soil loss rate. Geoderma.

[32] Bünemann E.K., Bongiorno G., Bai Z., Creamer R.E., De Deyn G., de Goede R., Fleskens L., Geissen V., Kuyper T.W., Mäder P. (2018). Soil quality—A critical review. Soil Biol. Biochem..

[33] Reinhart K.O., Vermeire L.T. (2016). Soil aggregate stability and grassland productivity associations in a northern mixed-grass prairie. PLoS ONE.

[34] Pham T.G., Nguyen H.T., Kappas M. (2018). Assessment of soil quality indicators under different agricultural land uses and topographic aspects in Central Vietnam. J. Soil Water Conserv..

[35] Haydu-Houdeshell C., Graham R.C., Hendrix P.F., Peterson A.C. (2018). Soil aggregate stability under chaparral species in southern California. Geoderma.

[36] De Paul Obade V., Lal R. (2013). Assessing land cover and soil quality by remote sensing and geographical information systems (GIS). Catena.

[37] Liu M., Han G., Zhang Q. (2019). Effects of soil aggregate stability on soil organic carbon and nitrogen under land use change in an erodible region in Southwest China. Int. J. Environ. Res. Public Health..

[38] Affandi N.F.L., Rusli S.H., Suhaini A.M., Baharulrazi N. (2018). Effect of pH on Growth Rate and Yield of Cucumis sativus. Chem. Eng..

[39] Tyler M., Hunter L., Steiner F., Roe D. (1987). Use of agricultural land evaluation and site assessment in Whitman County, Washington, USA. Environ. Manag..

[40] Li W., Wang D., Li H., Liu S. (2017). Urbanisation-induced site condition changes of peri-urban cultivated land in the black soil region of northeast China. Ecol. Indic..

[41] Araya A., Stroosnijder L. (2011). Assessing drought risk and irrigation need in northern Ethiopia. Agric. For. Meteorol..

[42] Worqlul A.W., Jeong J., Dile Y.T., Osorio J., Schmitter P., Gerik T., Srinivasan R., Clark N. (2017). Assessing potential land suitable for surface irrigation using groundwater in Ethiopia. Appl. Geogr..

[43] Sun J., Li Y.P., Suo C., Liu Y.R. (2019). Impacts of irrigation efficiency on agricultural water-land nexus system management under multiple uncertainties—A case study in Amu Darya River basin, Central Asia. Agric. Water Manag..

[44] Jiang R., Lu Q., Peng Z. (2018). A station-based rail transit network vulnerability measure considering land use dependency. J. Transp. Geogr..

[45] Castella J., Manh P.H., Kam S.P., Villano L., Tronche N.R. (2005). Analysis of village accessibility and its impact on land use dynamics in a mountainous province of northern Vietnam. Appl. Geogr..

[46] Nagendra H., Southworth J., Tucker C. (2003). Accessibility as a determinant of landscape transformation in western Honduras: Linking pattern and process. Landsc. Ecol..

[47] Ludeke A.K., Maggio R.C., Reid L.M. (1990). An analysis of anthropogenic deforestation using logistic regression and GIS. J. Environ. Manag..

[48] Liu Y., Xue J., Gui D., Lei J., Sun H., Lv G., Zhang Z. (2018). Agricultural Oasis Expansion and Its Impact on Oasis Landscape Patterns in the Southern Margin of Tarim Basin, Northwest China. Sustainability.

[49] Imbrenda V., Coluzzi R., Lanfredi M., Loperte A., Satriani A., Simoniello T. (2018). Analysis of landscape evolution in a vulnerable coastal area under natural and human pressure. Geomat. Nat. Hazards Risk.

[50] Wang Z., Chen J., Zheng W., Deng X. (2018). Dynamics of land use efficiency with ecological intercorrelation in regional development. Landsc. Urban Plan..

[51] Li E., Endter-Wada J., Li S. (2019). Dynamics of Utah’s agricultural landscapes in response to urbanisation: A comparison between irrigated and non-irrigated agricultural lands. Appl. Geogr..

[52] Chigbu U.E., Ntihinyurwa P.D., de Vries W.T., Ngenzi E.I. (2019). Why Tenure Responsive Land-Use Planning Matters: Insights for Land Use Consolidation for Food Security in Rwanda. Int. J. Environ. Res. Public Health.

[53] Li L. (2018). Towards a protocol on fair compensation in cases of legitimate land tenure changes: Input document for a participatory process. J. Chin. Gov..

[54] Kibblewhite M.G., Ritz K., Swift M.J. (2007). Soil health in agricultural systems. Philos. Trans. R. Soc. B.

